# A Multi-Level Study on the Anti-Lung Cancer Mechanism of Peiminine, a Key Component of *Fritillaria ussuriensis* Maxim.: Integrating Quality Analysis, Network Pharmacology, Bioinformatics Analysis, and Experimental Validation

**DOI:** 10.3390/ijms26083506

**Published:** 2025-04-09

**Authors:** Ziwen Yang, Shah Syed Faizan Ali, Xinhui Huang, Lin Wei, Yinze Zhong, Xuepeng Shi, Xiaotian Wu, Chunli Gan, Zhibin Wang, Chunjuan Yang

**Affiliations:** 1 Department of Pharmaceutical Analysis and Analytical Chemistry, College of Pharmacy, Harbin Medical University, Harbin 150000, China; ziwenyang0628@163.com (Z.Y.); fazanshah661@gmail.com (S.S.F.A.); huangxinhuiapply@163.com (X.H.); 15648571110@163.com (L.W.); zzz1170214@163.com (Y.Z.); sxp08031026@163.com (X.S.); wuxiaotian0918@126.com (X.W.); chunligan@126.com (C.G.); 2 Key Laboratory of Basic and Application Research of Beiyao, Ministry of Education, Heilongjiang University of Chinese Medicine, Harbin 150000, China; wzbmailbox@126.com

**Keywords:** peiminine, lung cancer, molecular docking, PI3K–Akt signaling pathway, network pharmacology

## Abstract

Globally, lung cancer is the primary cause of deaths associated with cancer; however, current therapies are costly and toxic, highlighting the need for novel treatments. Peiminine (Verticinone), a key bioactive compound derived from *Fritillaria ussuriensis* Maxim., has demonstrated diverse biological activities. However, the precise pharmacological mechanisms underlying its anti-lung cancer effects remain unclear. The objective of this study was to quantify the content of peiminine in *Fritillaria ussuriensis* Maxim. from different geographical regions using UHPLC-MS/MS and to elucidate the anti-lung cancer mechanisms of peiminine through network pharmacology, bioinformatics, and in vitro experiments. The content of peiminine in *Fritillaria ussuriensis* Maxim. from various regions was determined using UHPLC-MS/MS. Potential target genes associated with peiminine and lung cancer were systematically screened from multiple databases. To identify core genes, we set up a PPI (protein–protein interaction) network, followed by in-depth analyses of their corresponding target proteins. Survival analysis, molecular docking, and dynamics simulations were used to explore potential anti-cancer mechanisms. In vitro experiments on human H1299 NSCLC cells assessed peiminine’s anti-tumor activity and measured key gene transcription levels. UHPLC-MS/MS analysis revealed that *Fritillaria ussuriensis* Maxim. from Mudanjiang (Heilongjiang Province) exhibited the highest peiminine content. Network pharmacological analysis identified PIK3CG, SRC, JAK3, AKT2, and PRKCA as key potential targets of peiminine in lung cancer treatment. Molecular docking results demonstrated strong binding affinities between peiminine and PIK3CG, SRC, and JAK3; these results were further confirmed using molecular dynamics simulations. Survival analysis indicated that a high AKT2 and PRKCA expression correlated with bad prognosis in lung cancer patients. In vitro, peiminine inhibited H1299 cell viability and regulated genes involved in the PI3K–Akt pathway (PI3K, AKT, and PTEN) and apoptosis (Bcl-2, Bax), suggesting that it may induce its effects via PI3K–Akt pathway inhibition. Peiminine from *Fritillaria ussuriensis* Maxim. exhibits significant anti-lung cancer potential by targeting key genes such as PIK3CG, SRC, and JAK3, as well as by modulating the PI3K-Akt signaling pathway and apoptosis-related genes. These results lay a foundation for further investigations into peiminine as a potentially effective therapeutic option for treating lung cancer. Additionally, the identified targets (PIK3CG, SRC, JAK3, AKT2, and PRKCA) may function as possible biomarkers for predicting lung cancer prognosis and guiding personalized therapy.

## 1. Introduction

Cancer has emerged as one of the most significant global health challenges, representing the leading cause of death in many countries and posing a substantial barrier to increasing life expectancy worldwide [1]. According to the World Health Organization (WHO), cancer is the primary cause of mortality among individuals under 70 years old in 183 countries, with lung cancer being one of the most prevalent and deadly forms [2]. The development of cancer is a complex, multi-stage process involving initiation, promotion, and progression, each of which offers potential opportunities for pharmacological intervention to halt or reverse tumor growth. Despite advances in treatment modalities, including surgery, chemotherapy, radiotherapy, immunotherapy, and targeted therapies, significant challenges remain [3,4]. Drug resistance, particularly to chemotherapy and targeted therapies, has become a major obstacle, significantly reducing treatment efficacy [5]. Additionally, the delivery of targeted drugs is often inefficient due to poor bioavailability or tumor heterogeneity, further limiting their therapeutic potential [6]. The high cost of cancer medications exacerbates the financial burden on patients and healthcare systems, while chemotherapy and radiotherapy are frequently associated with severe side effects, such as myelosuppression and organ toxicity, which compromise patient outcomes and quality of life [7,8].

In light of these challenges, it is of great urgency that we search for treatment approaches in lung cancer. In recent years, natural compounds extracted from traditional Chinese medicine (TCM) have attracted growing interest due to their potential applications in cancer therapy. Unlike conventional therapies that primarily target cancer cells, TCM emphasizes the enhancement of the body’s immune system and overall health, thereby reducing toxic side effects. Many licensed anti-cancer drugs, such as vincristine, camptothecin, and paclitaxel, are derived from natural compounds with optimized structures [9]. Similarly, TCM has been widely recognized for its unique approach to cancer treatment, which focuses on restoring balance within the body rather than solely targeting cancer cells. This holistic approach significantly reduces toxic side effects, highlighting a key advantage of TCM [10,11].

Among the various medicinal herbs used in TCM, Fritillaria, a perennial herb of the Liliaceae family, has been used for over 2000 years for its therapeutic properties. In TCM, *Fritillaria ussuriensis* Maxim. primarily acts on the lung meridian and was traditionally used in cough-relieving and phlegm-resolving formulas. Fritillaria-based formulations, like cough syrups, are commonly used and have achieved international acclaim [12]. These formulations are prevalently found across China, Japan, and South Korea, and their annual sales surpass USD 70 million [13,14]. Recent studies have shown that the components of Fritillaria, particularly its alkaloids, exhibit significant cytotoxicity against cancer cells and may protect against lung damage [15,16]. These findings highlight Fritillaria’s potential as a natural source in lung cancer treatment.

Peiminine, a natural alkaloid that is present in roughly 13 species of Fritillaria, serves as the benchmark for determining the quality of *Fritillaria ussuriensis* Maxim. in the *Pharmacopoeia of the People’s Republic of China* [17]. This compound exhibits multiple pharmacological activities with promising therapeutic potential. Primarily recognized for its pulmonary protective effects, peiminine demonstrates efficacy in alleviating pulmonary fibrosis and LPS-induced lung injury [18,19]. Its anti-inflammatory properties further contribute to respiratory health management [20]. Notably, peiminine shows significant anti-cancer potential through multiple mechanisms. Studies have demonstrated that it can suppress the growth of urothelial bladder cancer cells. This inhibition is achieved through the induction of cell death and the arrest of the cell cycle [21]. In breast cancer treatment, peiminine shows synergistic actions in combination with Adriamycin by suppressing the PI3K-Akt pathway and triggering ferroptosis [22]. Although preliminary studies suggest specific activity against human cancer cells, the precise mechanisms underlying peiminine’s anti-cancer effects remain incompletely understood [23].

Network pharmacology, a newly emerging methodology that fuses traditional pharmacology, molecular biology, and bioinformatics, provides a comprehensive view of how drugs work. It achieves this by examining the intricate relationships between drugs, numerous targets, and biological pathways. This approach is particularly well suited to the study of TCM, which often exerts its effects through multi-target mechanisms [24,25,26]. In the present research, our objective was to pinpoint the potential targets of peiminine for treating lung cancer using network pharmacology and molecular docking methods. Subsequently, in vitro experiments were conducted to verify its cytotoxic impacts and regulatory mechanisms.

To address these challenges, this study employed a multi-level research approach to systematically explore the anti-cancer effects of peiminine and its molecular mechanisms. Network pharmacology was utilized to identify potential targets of peiminine in NSCLC treatment by constructing complex networks that depict the relationships between peiminine, genes, and biological pathways. Molecular docking, which simulates the interaction between peiminine and its potential protein targets, provided an initial validation of these interactions. To further validate the molecular docking outcomes, molecular dynamics simulations were conducted to study the dynamic behavior of ligand–protein complexes over time, offering deeper insights into their stability and interaction patterns. Finally, the findings of network pharmacology and prognostic models were validated through in vitro experiments. This study sought to develop an effective and low-toxicity treatment, addressing the urgent need for innovative strategies in cancer therapy.

## 2. Results

### 2.1. Determination of the Content of Peiminine in Fritillaria ussuriensis Maxim. Using UPLC-MS/MS

#### 2.1.1. Method Validation Results

**Figure 1:**
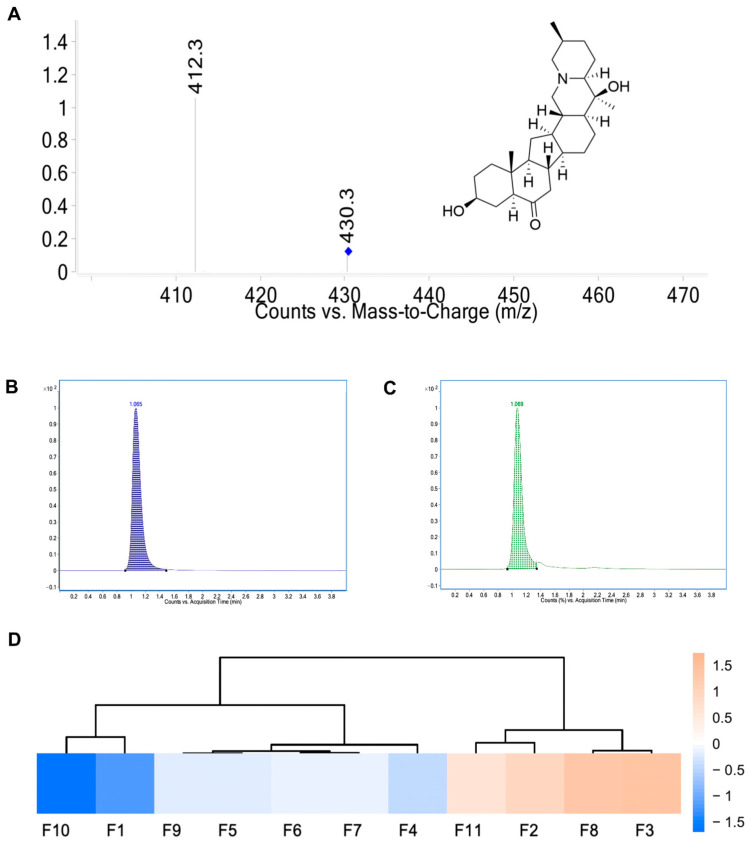
(**A**) Product ion mass sepctra and structure of peiminine. (**B**) Representative chromatograms of peiminine reference solution. (**C**) Representative chromatogram of test solution of samples. (**D**) Heat map of peiminine content in *Fritillaria ussuriensis* Maxim. from different regions.

Figure 1A illustrates the analytical characterization of peiminine, including its product ion mass spectrum and chemical structure. As shown in Figure 1B and Figure 1C, the identical retention time of chromatographic peaks between the reference standard and test solution indicates excellent method specificity. The regression equation for peiminine (y = 512.2x + 45323) achieved a determination coefficient (R^2^ = 0.988) demonstrating strong linearity within the tested concentration range. The RSD of chromatographic peak areas for peiminine was 2.51%, confirming good precision. Additionally, stability testing of the fritillary extract test solution and spiked recovery experiments met acceptance criteria, further validating the method’s reliability. Repeatability testing yielded an RSD of 0.70% for peiminine in the extract, ensuring its reproducibility. With a limit of quantification (LOQ) of 0.52 μg/mL (S/N = 15.8), the established method is suitable for quantifying peiminine in samples of diverse geographical origins.

#### 2.1.2. Sample Analysis

The established UHPLC-MS/MS method was applied to determine the peiminine content in *Fritillaria ussuriensis* Maxim. extract using the external standard method. The geographical distribution of peiminine content across *Fritillaria ussuriensis* Maxim. samples is visualized in a heat map (Figure 1D). Table 1 indicates that the content of peiminine in *Fritillaria ussuriensis* Maxim. from different producing areas varies. Among them, *Fritillaria ussuriensis* Maxim. from the Mudanjiang area in Heilongjiang Province has the highest content of peiminine, at 1134.64 µg/g in the extract.

### 2.2. Target Screening for Peiminine and Lung Cancer

**Figure 2:**
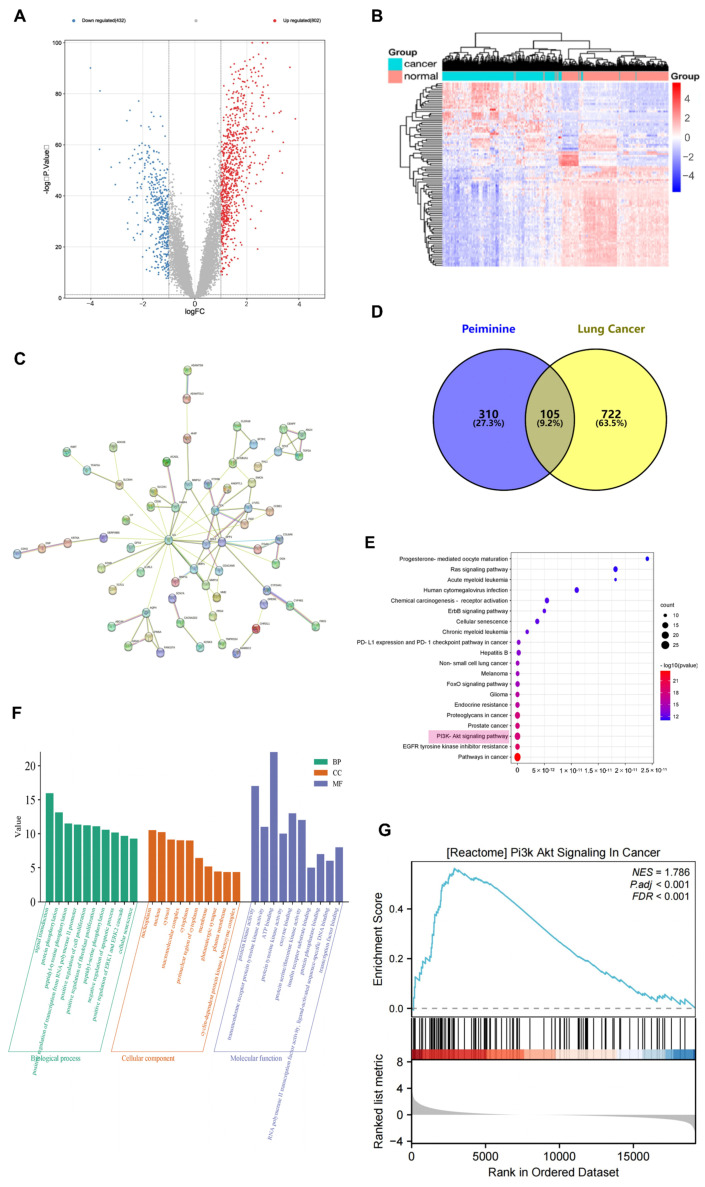
Related targets of lung cancer and peiminine. (**A**) The volcano map of differentially expressed genes. (**B**) Gene expression heat map. (**C**) PPI network of top 100 differentially expressed genes. (**D**) Venn diagram. (**E**) KEGG pathway. (**F**) GO analysis. (**G**) GSEA of the PI3K–Akt signaling pathway in lung cancer.

By searching a series of databases, we found 415 potential targets of peiminine by retrieving lung cancer-related targets from the disease database. The screening criteria of the Genecards and DisGeNet databases are, respectively, a relevance score ≥ 50 and a score_dge ≥ 0.1. After the GSE151103 data set was processed using the GEO2R online tool, 1195 differentially expressed genes were found via screening with *p*-value and logFC criteria, including 432 downregulated genes and 802 upregulated genes. A volcano plot (Figure 2A) showcases the genes with differential expression. Genes that are upregulated are colored red, while those that are downregulated are in blue, which effectively emphasizes the notable alterations in gene expression levels. The top 100 differentially expressed genes were selected, and a gene expression heat map (Figure 2B) was drawn; its network was drawn using cystoscope (Figure 2C). The top 100 differentially expressed genes were combined with the target of peiminine to find 105 overlapping targets (Figure 2D).

### 2.3. PPI Network Analysis

**Figure 3:**
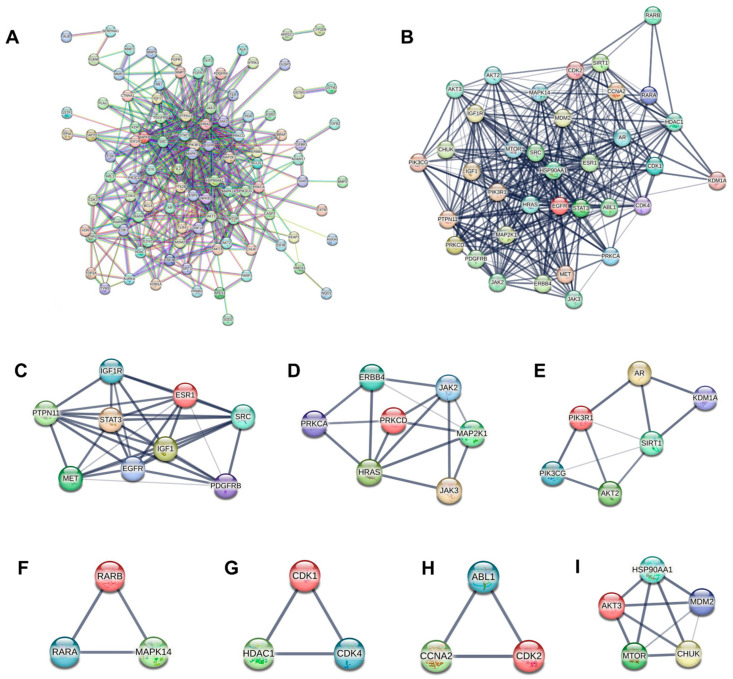
The PPI network. (**A**) A total of 105 targets. (**B**) PPI network of the 36 core targets. (**C**–**I**) Seven modules selected from the PPI network.

Protein–protein interaction (PPI) networks, representing connections between proteins, are of great significance in the comprehension of cellular functions. In the present research, the STRING online platform was utilized to conduct an analysis of the PPI network. A comprehensive exploration was carried out on the PPI network consisting of 105 targets (shown in Figure 3A). When the confidence level was set to 0.9, we generated a PPI network that had 105 nodes and 465 edges. Next, we loaded the PPI network into Cytoscape software (v3.9.1; http://www.cytoscape.org, accessed on 10 June 2024). By utilizing the MCODE plugin within Cytoscape, seven distinct modules were successfully extracted from the PPI network (Figure 3C–I). These modules collectively contained 36 genes, which were then regarded as core genes. To further explore the functional significance of these core genes, a sub-network of the core genes was constructed (Figure 3B), enabling an in-depth analysis of their interactions and potential roles in the biological processes under study.

### 2.4. GO Analysis, KEGG Analysis, and GSEA

To obtain a more in-depth understanding of the functional roles of, and pathways involved in, the core genes in lung cancer, we used the DAVID tool to conduct Gene Ontology (GO) and Kyoto Encyclopedia of Genes and Genomes (KEGG) pathway enrichment analyses. A total of 302 biological processes (BPs), 33 cellular components (CCs), 71 molecular functions (MFs), and 137 KEGG pathways were identified. To visually display the top 20 enriched terms for each GO category and KEGG pathway, we created bubble charts (Figure 2E,F). The results show that the core genes are primarily involved in signal transduction, protein phosphorylation, peptidyl–tyrosine phosphorylation, and the positive regulation of transcription from the RNA polymerase II promoter. These functions are enabled by mechanisms such as protein kinase activity, transmembrane receptor protein tyrosine kinase activity, ATP binding, protein tyrosine kinase activity, and enzyme binding. Among the 137 KEGG pathways, the most significantly enriched included pathways related to cancer, EGFR tyrosine kinase inhibitor resistance, the PI3K–Akt signaling pathway, prostate cancer, proteoglycans in cancer, and endocrine resistance. Notably, 21 core genes were associated with the PI3K–Akt signaling pathway. As shown in Figure 2G, GSEA revealed a significant enrichment of the PI3K–AKT signaling pathway in lung cancer (NES = 1.786; *p* < 0.001; FDR < 0.001), a finding that is consistent with the predictions of the network pharmacology analysis. This implies that peiminine might primarily exert its anti-cancer impacts via the PI3K–Akt signaling pathway.

### 2.5. Molecular Docking Results

**Figure 4:**
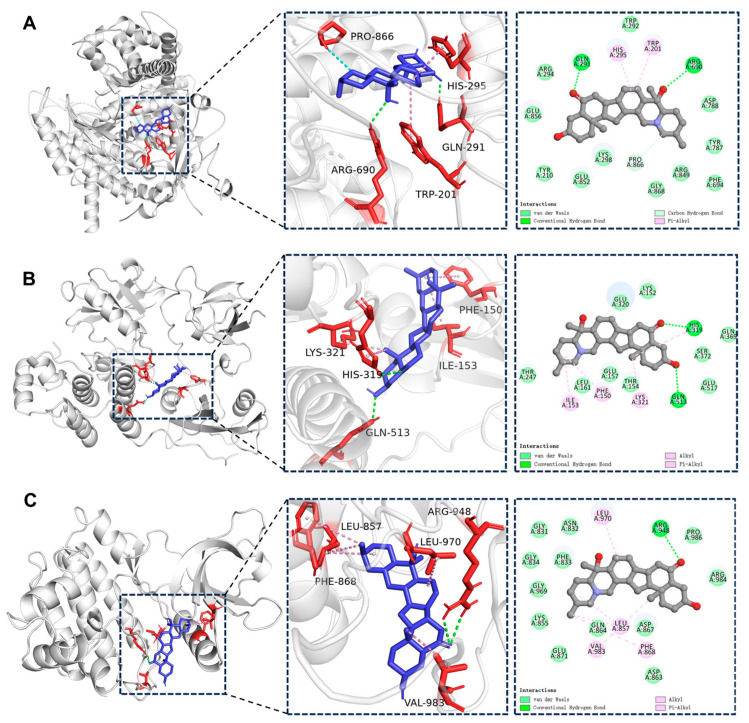
The 3D interaction between peiminine and the three genes. (**A**) PIK3CG; (**B**) SRC; (**C**) JAK3.

When the docking score is lower than 0 kcal/mol, it means that the component can spontaneously bind to the target. A score below −4.25 kcal/mol indicates a good docking affinity, and when the score is below −7 kcal/mol, it shows a strong docking affinity (45). As shown in Table 2, peiminine demonstrated good docking activity with 36 target genes, with the lowest binding energies being observed for PIK3CG, SRC, and JAK3. The binding interactions between peiminine and these three genes are illustrated in Figure 4. The docking binding energy between PIK3CG and peiminine was −10.1 kcal/mol (<−7 kcal/mol), indicating strong docking affinity. This implies that peiminine could efficiently suppress the activity of PIK3CG, which is a crucial protein within the PI3K–Akt signaling pathway. The binding is primarily mediated by hydrogen bonds and hydrophobic interactions. Specifically, peiminine forms hydrogen bonds with GLN-291 and ARG-690 on PIK3CG, as well as unconventional hydrogen bonds with PRO-866. Additionally, hydrophobic interactions include Pi–alkyl bonds with TRP-201 and HIS-295. Similarly, the docking binding energy between SRC and peiminine was −10.1 kcal/mol (<−7 kcal/mol), also indicating a strong docking affinity. The interaction is driven by hydrogen bonds and hydrophobic forces.

Peiminine forms hydrogen bonds with HIS-319 and GLN-513 on SRC, while hydrophobic interactions include alkyl bonds with ILE-153 and LYS-321, as well as Pi–alkyl bonds with PHE-150 and HIS-319. For JAK3, the docking binding energy with peiminine was −9.9 kcal/mol (<−7 kcal/mol), reflecting a strong docking affinity. The binding is stabilized by hydrogen bonds and hydrophobic interactions. Peiminine forms two hydrogen bonds with ARG-948 on JAK3, while hydrophobic interactions include alkyl bonds with VAL-983, LEU-970, and LEU-857, as well as Pi–alkyl bonds with PHE-868.

### 2.6. MD Analysis

**Figure 5:**
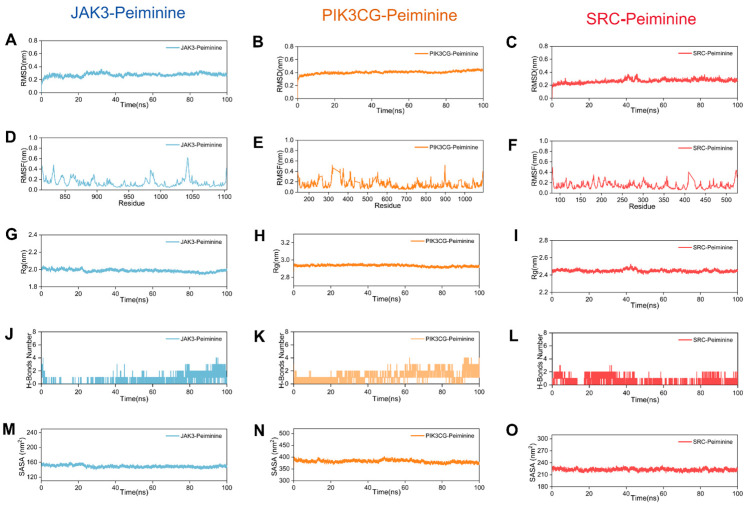
Molecular dynamics simulation of three complexes (JAK3 in blue, PIK3CG in orange, and SRC in red). (**A**–**C**) The RMSD curves of JAK3, PIK3CG, and SRC proteins with peiminine. (**D**–**F**) The RMSF curves of the complexes of JAK3, PIK3CG, and SRC proteins with peiminine. (**G**–**I**) The Rg curves of the complexes of JAK3, PIK3CG, and SRC proteins with peiminine. (**J**–**L**) The fluctuation curve of the number of hydrogen bonds formed between the JAK3, PIK3CG, and SRC proteins with peiminine. (**M**–**O**) The SASA curves of the complexes of the JAK3, PIK3CG, and SRC proteins with peiminine.

The structural integrity of Peiminine-bound JAK3, PIK3CG, and SRC complexes was systematically evaluated using multiple biophysical metrics. Root Mean Square Deviation (RMSD) trajectories (Figure 5A–C) demonstrated exceptional stability, with all systems maintaining deviations below 1 nm throughout the simulation. This tight conformational consistency (<1 nm) underscores the robust binding interactions between Peiminine and its targets. Residue-level flexibility was probed via Root Mean Square Fluctuation (RMSF) analysis (Figure 5D–F). Notably, amino acid residues in all three complexes exhibited minimal fluctuations (<1 nm), indicating that Peiminine binding imposes structural constraints without inducing significant dynamic perturbations. Radius of gyration (Rg) measurements (Figure 5G–I) further validated the compactness of the complexes. The JAK3, PIK3CG, and SRC systems maintained stable Rg values of ~2.0 nm, 2.9 nm, and 2.5 nm, respectively, confirming the preservation of native-like structural compaction during the simulation. Hydrogen bond analysis (Figure 5J–L) revealed persistent intermolecular interactions, with 1–2 hydrogen bonds retained consistently across all trajectories. This hydrogen bonding network likely contributes to the observed stability by reinforcing ligand–protein interactions.

**Figure 6:**
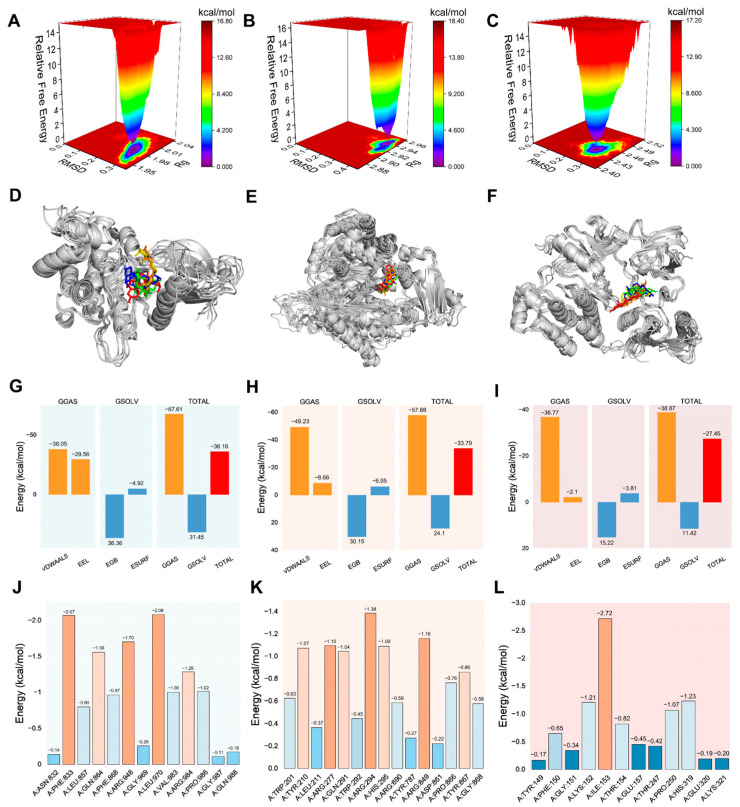
Molecular dynamics simulation. (**A**–**C**) Free energy distribution diagrams of the JAK3, PIK3CG, and SRC complexes with peiminine. (**D**–**F**) Structural comparisons at 0, 25, 50, 75, and 100 ns show peiminine’s positions (red, green, blue, yellow, and orange, respectively). (**G**–**I**) Average binding free energies of the complexes, calculated using MM/GBSA. Key terms include Van der Waals (VDWAALS), electrostatic (EEL), polar solvation (EGB), non-polar solvation (ESURF), molecular mechanics (GGAS), solvation (GSOLV), and total binding free energy (TOTAL). (**J**–**L**) Energy contributions of key residues in the JAK3, PIK3CG, and SRC proteins binding to peiminine. Critical binding contributions—JAK3: LEU-970 (−2.08 kcal/mol), PHE-833 (−2.07 kcal/mol), and ARG-948 (−1.70 kcal/mol); PIK3CG: ARG-284 (−1.38 kcal/mol), ARG-849 (−1.16 kcal/mol), and ARG-277 (−1.10 kcal/mol); SRC: ILE-153 (−2.72 kcal/mol).

Collectively, these complementary metrics (RMSD, RMSF, Rg, and hydrogen bonding) converge to support the conclusion that Peiminine forms stable complexes with JAK3, PIK3CG, and SRC, with no evidence of conformational destabilization. In summary, the combined analysis of RMSD, RMSF, Rg, and hydrogen bonding demonstrates that the JAK3, PIK3CG, and SRC complexes with Peiminine exhibit excellent structural stability, with Peiminine binding having a minimal impact on protein conformations. The solvent-accessible surface area (SASA) is a key indicator of protein folding and stability, with stable conformations typically showing consistent SASA profiles. Lower and stable SASA values suggest tightly folded and stable conformations during binding. As shown in Figure 5M–O, the SASA trajectories for the JAK3, PIK3CG, and SRC complexes with Peiminine exhibit minimal fluctuations, with variation ranges of approximately 160, 400, and 230 nm^2^, respectively, indicating strong structural stability. To further assess stability, the relative Gibbs free energy was calculated using RMSD and Rg values, and a three-dimensional free-energy landscape was constructed (Figure 6A–C). Here, dark purple and blue regions represent the most stable, low-energy conformations, while red and yellow areas indicate less stable states. The free-energy diagrams for the three complexes reveal a single, concentrated minimum-energy cluster, demonstrating that the JAK3, PIK3CG, and SRC complexes with Peiminine exhibit excellent stability.

### 2.7. Survival, Expression, and Correlation Analysis

**Figure 7:**
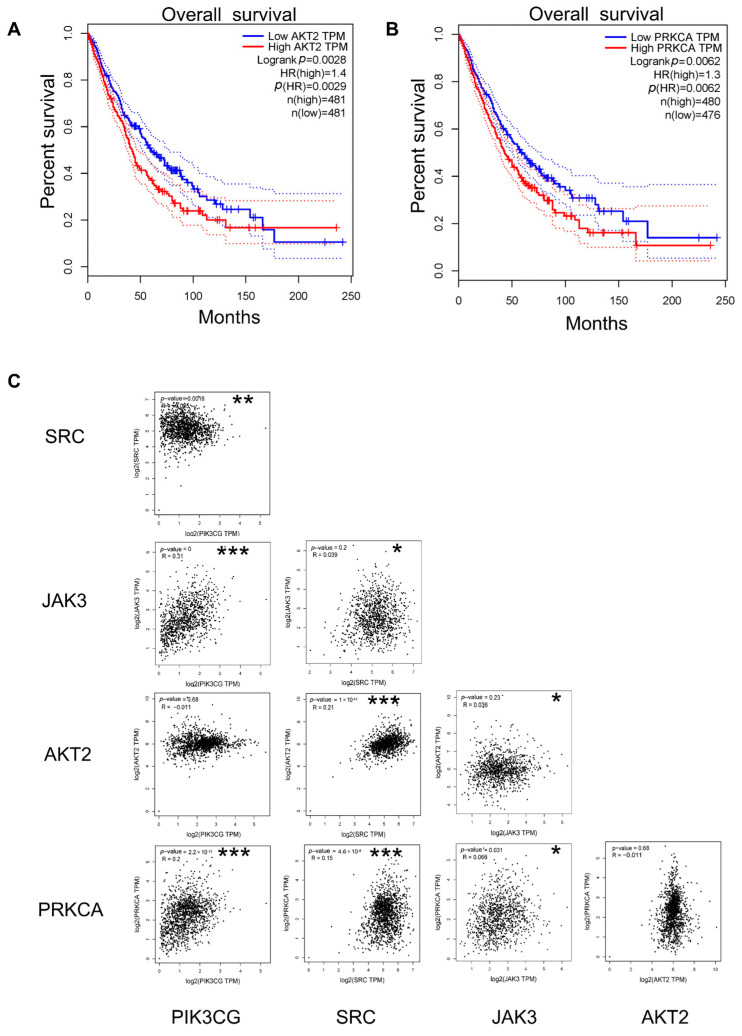
Survival, expression, and correlation analysis. (**A**) Kaplan–Meier survival curve of AKT2. (**B**) Kaplan–Meier survival curve of PRKCA. (**C**) Correlation analysis of PIK3CG, SRC, JAK3, AKT2, and PRKCA. * for *p* value less than 0.05, ** for *p* value less than 0.01, and *** for *p* value less than 0.001.

Using the GEPIA online tool, 36 key genes were categorized into high- and low-expression groups based on the median expression levels, and their prognostic significance in lung cancer was evaluated. Survival analysis revealed that only patients with a high expression of the AKT2 and PRKCA genes among the 36 genes exhibited poor prognosis (Figure 7A,B). These findings indicate that AKT2 and PRKCA serve as reliable predictors of patient survival outcomes. Correlation analysis (Figure 7C) demonstrated positive relationships between PIK3CG and JAK3, PIK3CG and PRKCA, SRC and AKT2, SRC and PRKCA, and JAK3 and PRKCA. Conversely, negative correlations were observed between PIK3CG and SRC, as well as between JAK3 and AKT2. No significant relationships were found between other gene combinations.

### 2.8. Effects of Peiminine on the Viability of H1299 Cells

**Figure 8:**
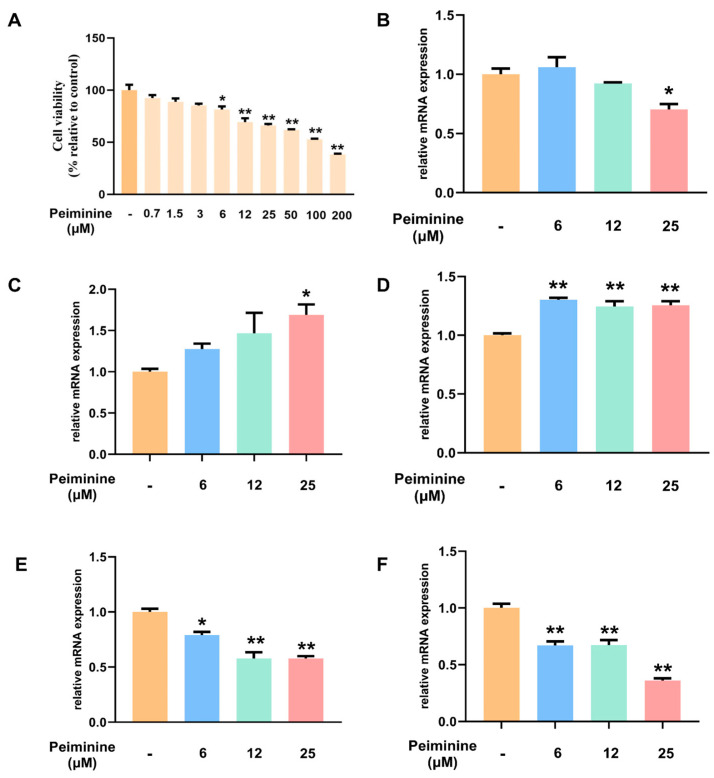
The effect of peiminine in H1299 cells. (**A**) Cell viability of H1299 cells with different doses of peiminine. (**B**–**F**) The transcription levels of key genes after peiminine administration. Orange for untreated group, blue for 6 μM peiminine treatment group, green for 12 μM peiminine treatment group, and red for 25 μM peiminine treatment group. (**B**) PI3K. (**C**) PTEN. (**D**) Bax. (**E**) Bcl-2. (**F**) AKT. * *p* < 0.05 and ** *p* < 0.01 versus group without peiminine treatment.

To predict the therapeutic potential of peiminine in lung cancer, network pharmacology and molecular docking approaches were employed. To validate these predictions, in vitro experiments were conducted using the H1299 cell line. The impact of peiminine on H1299 cell viability was assessed after 24 h of treatment. As shown in Figure 8A, compared to the control group, increasing concentrations of peiminine (ranging from 0.7 μM to 200 μM) led to a dose-dependent reduction in cell viability. Significant decreases in viability were observed at concentrations of 6 μM, 12 μM, 25 μM, 50 μM, 100 μM, and 200 μM (*: *p* < 0.05; **: *p* < 0.01). The half-maximal inhibitory concentration (IC50) was determined by plotting the data on a logarithmic scale, revealing an IC50 value of 97.4 μM for peiminine in H1299 cells. Subsequent experiments were conducted using peiminine concentrations of 6 μM, 12 μM, and 25 μM.

### 2.9. Transcriptional Regulation of the PI3K–Akt Pathway and Apoptosis Pathway by Peiminine in H1299 Cells

After peiminine administration, the mRNA expression levels of PI3K, AKT, PTEN, Bax, and Bcl2 in H1299 cells were examined using qPCR. As shown in Figure 8, compared to the control group, treatment with peiminine led to a decrease in PI3K mRNA expression at concentrations of 12 μM and 25 μM, with the reduction at 25 μM being statistically significant (*p* < 0.01). Concurrently, peiminine treatment induced a dose-dependent decrease in AKT mRNA expression, with a significant downregulation (*p* < 0.05 at 6 μM and *p* < 0.01 at 12 μM and 25 μM). Regarding the apoptosis-related genes, Bax mRNA expression was significantly upregulated at all tested peiminine concentrations (*p* < 0.01 for all), while Bcl-2 mRNA expression was downregulated, with significant decreases noted at 12 μM and 25 μM (*p* < 0.01) and at 6 μM (*p* < 0.05). Collectively, these results indicate that peiminine exerts a significant impact on both the PI3K–AKT signaling pathway and apoptosis-related gene expression in H1299 cells. These findings imply that peiminine may have potential anti-tumor activity by downregulating the PI3K–Akt signaling pathway and upregulating pro-apoptotic genes, leading to reduced cell proliferation and increased apoptosis in cancer cells, providing a basis for further investigation into its mechanism of action and potential applications in cancer treatment.

## 3. Discussion

Lung cancer can be generally classified into two main types—non-small-cell lung cancer (NSCLC) and small-cell lung cancer (SCLC). NSCLC constitutes 80% to 85% of all lung cancer cases [27]. In the case of patients with locally advanced non-small-cell lung cancer (NSCLC) who are not suitable for surgery, the principal treatment alternatives consist of chemotherapy and radiation therapy. Current protocols typically involve a 6-week course of chest radiation combined with dual chemotherapy using cisplatin. However, these treatments are associated with low survival rates and significant physical side effects, which severely compromise patients’ quality of life [28,29]. Considering these challenges, it is of the utmost urgency to explore and develop innovative and more efficacious therapeutic agents in order to enhance treatment outcomes for patients suffering from lung cancer.

With an extensive cultivation history spanning centuries, *Fritillaria ussuriensis* Maxim. is primarily distributed across Heilongjiang, Jilin, and Liaoning provinces in China, alongside Russia’s Far Eastern coastal zones and the Korean Peninsula [13]. According to the *Chinese Pharmacopoeia* (2020 Edition), its efficacy in “clearing heat, moistening the lungs, and resolving phlegm” is well documented. Studies on *Fritillaria ussuriensis* Maxim. originate from its millennia-long history of clinical application, particularly in treating pulmonary diseases [30]. In this study, we identified that *Fritillaria ussuriensis* Maxim. sourced from Mudanjiang (Heilongjiang Province, China) contains the highest levels of peiminine. This finding not only provides critical evidence for refining the quality standards of *Fritillaria ussuriensis* Maxim. but also facilitates the selection of raw materials from high-quality producing areas, which will help reduce R&D costs, enhance efficiency, and accelerate the clinical translation of peiminine [31].

Our research identified 105 potential therapeutic targets of peiminine in lung cancer through the integration of multiple databases. To further elucidate its role in lung cancer treatment, a protein–protein interaction (PPI) network was constructed, and 36 core genes that potentially mediate the therapeutic effects of peiminine were identified. Through functional enrichment analysis with the use of Kyoto Encyclopedia of Genes and Genomes (KEGG) and Gene Ontology (GO), it was revealed that these core genes play roles in crucial biological processes, including signal transduction, protein phosphorylation, and the regulation of transcription. Key pathways associated with these genes include the PI3K-Akt signaling pathway, EGFR tyrosine kinase inhibitor resistance, and pathways in cancer, among others. The PI3K–AKT signaling pathway ranks among the most commonly dysregulated signaling pathways in human cancers [32]. Furthermore, Gene Set Enrichment Analysis (GSEA) identified significant enrichment of the PI3K–AKT signaling pathway in lung cancer tissues. These results indicate that peiminine might produce its anti-cancer effects through the PI3K–AKT signaling pathway.

Molecular docking studies further identified three core genes—PIK3CG, SRC, and JAK3—with the lowest binding energy to peiminine, indicating strong interactions. Molecular dynamics simulations were conducted to validate the stability of the complexes formed between peiminine and these proteins. We systematically evaluated the dynamic behavior of these complexes using multiple biophysical metrics including RMSD, RMSF, Rg, and hydrogen bond analysis, which collectively demonstrated stable interactions and conformational compactness. Complemented by survival analysis, identifying AKT2 and PRKCA as prognostic markers significantly correlated with lung cancer patient overall survival, we thereby prioritized these five genes as potential therapeutic targets for peiminine. While experimental validation is needed, the integration of structural dynamics data with clinical outcomes establishes a framework, underscoring Peiminine’s multi-targeted therapeutic potential.

Notably, the predicted targets of peiminine identified through multi-level studies serve as core components or regulators of the PI3K–Akt signaling pathway: PIK3CG functions as a catalytic subunit initiating signaling cascades driving malignant cell growth and survival [33,34]; SRC acts as a recognized inducer of the PI3K–Akt pathway [35,36]; and additional complexity arises from crosstalk mechanisms, including JAK3’s modulation of the JAK–STAT/PI3K–AKT axis [37] and PRKCA’s regulation of AKT phosphorylation [38]. Collectively, these interactions suggest broader network-level intervention by peiminine. Current research is focusing on experimentally verifying peiminine’s regulatory effect on the PI3K–AKT signaling pathway, while future studies will prioritize elucidating the detailed mechanisms underlying its anti-lung cancer efficacy and identifying clearer therapeutic targets.

Given the epidemiological predominance of NSCLC, our research specifically focused on this subtype to establish its clinical relevance. The H1299 cell line, derived from the large-cell carcinoma subtype of NSCLC in a metastatic lymph node, is widely recognized as a standardized model for drug screening studies in NSCLC [39,40]. To validate our theoretical findings, we conducted in vitro experiments demonstrating that peiminine significantly inhibited the proliferation of H1299 cells in a dose-dependent manner. Quantitative PCR (qPCR) analysis revealed that peiminine downregulates the mRNA expression of PI3K and Akt while upregulating PTEN, which is a negative regulator of the PI3K–Akt pathway. The emerging evidence chain demonstrates remarkable coherence between computational predictions and experimental validation. This inhibition of Akt activation suppresses downstream pro-survival and pro-proliferation signals, effectively curbing cancer cell growth. The dysregulation of apoptosis is a hallmark of cancer, and inducing apoptosis in cancer cells is a well-established therapeutic strategy. Consistent with this, we observed that peiminine treatment upregulated the pro-apoptotic gene Bax and downregulated the anti-apoptotic gene Bcl-2, further supporting its role in apoptosis induction via PI3K–Akt signaling pathway inhibition. These findings are in line with previous studies, such as the work of Qin et al., which demonstrated that the suppression of the PI3K/Akt pathway induces apoptosis in NSCLC [41]. Despite these promising results, our study has certain limitations. We prioritized the development of novel natural anti-tumor agents with well-defined molecular targets, though their broad applicability to other sub types of lung cancer requires further investigation in future studies. To validate these findings and evaluate the safety and efficacy of peiminine in human patients, in vivo studies and clinical trials are also required. These future studies may uncover a promising therapeutic strategy to combat this challenging disease.

Cisplatin remains a first-line chemotherapeutic agent for lung cancer; it was approved by the U.S. Food and Drug Administration in 1978 [42]. This study proposes peiminine as a novel lung cancer therapy, contrasting with cisplatin’s limitations: severe nephrotoxicity/neurotoxicity and tumor resistance via drug efflux pumps/DNA repair [43,44]. As the primary bioactive compound in *Fritillaria ussuriensis* Maxim., peiminine boasts centuries of safe dietary use (e.g., “Bei Mu Xue Li Geng”). Combined with the established food-grade application system of *Fritillaria ussuriensis* Maxim., peiminine inherits the safety characteristics of traditional dietary recipes. Peiminine is expected to develop into an innovative drug for the treatment of lung cancer, bridging TCM and modern precision oncology and offering a safer therapeutic strategy.

In summary, the results of this research indicate that peiminine exhibits remarkable anti-lung cancer properties by suppressing the PI3K–Akt signaling pathway with multi-targets. These discoveries offer novel perspectives on potential drug targets and treatment approaches especially for the NSCLC sub-type. Future research should address the limitations identified in this study, with the goal of advancing peiminine’s development as a therapeutic agent and offering more comprehensive insights into its anti-cancer mechanisms.

## 4. Materials and Methods

### 4.1. Determination of Peiminine Content in Fritillaria by UHPLC–MS/MS Method

#### 4.1.1. Preparation of Reference Substance Solution

Accurately weigh 0.01 g of peiminine reference substance, dissolve it in methanol in a 5 mL volumetric flask, and prepare standard stock solutions with a concentration of 2000.00 μg/mL each. According to the experimental requirements, the secondary and tertiary standard stock solutions should be further prepared by dilution with methanol.

#### 4.1.2. Preparation of Test Solution

Precisely weigh 2.0 g (accurate to 0.1 mg) of powdered *Fritillaria ussuriensis* Maxim. sample and place it into a 50 mL conical flask. Add 4 mL of ammonia water, seal the flask, and let it stand for 1.5 h for immersion. Precisely measure and add 40 mL of a chloroform–methanol mixed solution with a volume ratio of 4:1, mix well, and sonicate for 1 h. Filter the sample into a 50 mL round-bottom flask. Rinse with 15 mL of the mixed solution and transfer all the solution into the round-bottom flask. Use a rotary evaporator to evaporate the solution to near dryness. Add 5 mL of methanol to redissolve the residue and filter the solution for the sample solution.

#### 4.1.3. UHPLC–MS/MS: Instruments and Conditions

Analysis was conducted using an Agilent 1290 UHPLC system (Agilent Technologies, Santa Clara, CA, USA) combined with an Agilent 6430 triple quadrupole mass spectrometer equipped with an ESI source interface. A Waters HSS T3 column was used for quantification.

#### 4.1.4. Method Validation

Our method validation strictly followed FDA guidelines. The specific methods can be referred to in Supplementary Materials.

#### 4.1.5. Content Determination

The test sample solutions were prepared as in Section 4.1.2. The content of Peiminine in *Fritillaria ussuriensis* Maxim. samples was determined under the UHPLC-MS/MS conditions in Section 4.1.3.

### 4.2. Network Pharmacology

#### 4.2.1. Target Collection for Peiminine

Public databases (Pharmmapper, SwissTargetPrediction, SuperPRED, SEA, targetnet) were used to predict peiminine’s potential targets in Homo sapiens. The UniProt database was used to standardize the target gene list and remove duplicates.

#### 4.2.2. Target Collection for Lung Cancer from Databases

We utilized the keywords “lung cancer” and “carcinoma of the lungs” to search two public databases, namely, DisGeNET (https://disgenet.com/, accessed on 10 June 2024) with a cut-off value of “score_gda > 0.1” and GeneCards (https://www.genecards.org, accessed on 10 June 2024), with only genes with correlation scores higher than 30 being considered. This search was carried out to identify key targets associated with lung cancer in the “Homo sapiens” species. Afterwards, we gathered information on the target genes. The integrated list of these genes was then uploaded to UniProtKB to acquire their gene names and IDs. Subsequently, we eliminated any duplicate entries from the list.

#### 4.2.3. Target Collection for Lung Cancer from Expression Databases

GSE 151103 is a microarray data set screened from the NCBI GEO database retrieval data set to identify differentially expressed genes in lung cancer. GSE151103 is composed of 172 normal samples and 188 lung cancer samples (360 samples in total). We used GEO2R’s built-in methods (*t*-test, Benjamini, and Hochberg) and force normalization to find differentially expressed genes (DEGs) between lung cancer patients and controls, eliminating data redundancy and errors. A volcano plot showed significant up/downregulated genes. We filtered genes with adjusted *p* < 0.01 and log2FC ≥ 2 as DEGs for further study. SRplot was used to plot a heat map of two data sets, and the network of the top 100 differentially expressed genes obtained was plotted using Cytoscape. We integrated disease target database targets and top 100 DEGs and created a Venn diagram to find genes shared by peiminine and lung cancer targets.

#### 4.2.4. PPI Network Construction

We employed the online STRING tool (version 11.5, http://string-db.org, accessed on 10 June 2024) to construct a protein–protein interaction (PPI) network for the differentially expressed genes (DEGs) obtained from the data sets. An interaction score threshold of 0.9 was set as the minimum requirement, and nodes with no interactions with other nodes were removed from the network. By analyzing the functional interactions between proteins, we can gain insights into the underlying biological mechanisms. The PPI network generated by STRING was then imported into Cytoscape (version 3.9.1; http://www.cytoscape.org, accessed on 10 June 2024) to pinpoint hub genes and key modules. Since these networks follow a scale-free distribution, we evaluated the biological significance of genes through degree centrality. In Cytoscape, we carried out an analysis of network parameters such as degree, closeness, and betweenness. To identify hub genes within the network, we set a degree cut-off value greater than 10. Additionally, using the MCODE plugin, we identified intersected clusters with specific parameters: a Node cutoff score of 0.2, a K-score of 2, and a Max Depth of 100.

#### 4.2.5. Pathway and Functional Enrichment Analysis

We used DAVID (v6.8, https://david.ncifcrf.gov/, accessed on 10 June 2024) for GO and KEGG enrichment analysis to find target proteins’ functions and cancer-related pathways. We selected enriched GO terms and pathways with FDR < 0.01 for visualization and plotted them as bubble graphs.

### 4.3. GSEA

In order to explore the biological pathways involved in prognostic genes, the mRNA data sets of TCGA-LUAD and TCGA-LUSC (including tumor tissue and adjacent normal tissue) were downloaded from the TCGA database (https://portal.gdc.cancer.gov/, accessed on 16 January 2025). After integrating the samples, the R package “limma” was used to perform differential analysis on tumor tissue vs. normal tissue (Cancer vs. Normal), and log2FC was calculated. We sorted log2FC from large to small and then used the R package “clusterProfiler” to perform GSEA pathway enrichment analysis to visualize the signaling pathways of interest. The reference gene set is c2.cp.all.v2022.1.Hs.symbols.gmt in The Molecular Signatures Database (MSigDB) (http://www.gsea-msigdb.org/gsea/msigdb/index.jsp, accessed on 16 January 2025).

### 4.4. Molecular Docking

In order to forecast the interaction between the hub genes and peiminine, we carried out molecular docking. The PDB structures of 36 hub genes were fetched from the UniProt and RCSB databases [45]. Meanwhile, the 3D structure of peiminine was acquired from PubChem and transformed into the PDB format with the help of Open Babel [46]. Using Pymol, the water molecules and native ligand groups within the protein structure were eliminated. Subsequently, Pymol predicted the active site that would allow for the most effective ligand binding [47], which then predicted the active site for the most efficient ligand binding. Subsequent protein processing and molecular docking analyses were executed in AutoDock Tools, with individual docking simulations conducted for each target protein [48]. Finally, visualization was performed using Pymol2.5 and Discovery Studio 2019 [49].

### 4.5. Molecular Dynamics Simulation

Using Gromacs version 2024.4, we performed molecular dynamics simulations on the complexes of proteins and Peiminine. For the protein, we used the Amber14sb force-field for description, and for the ligand, the Gaff2 force-field was used for modeling. We used the TIP4P water model to solvate the system within a 1.2 nm periodic boundary water box. Long-range electrostatic interactions were computed via the PME approach, and ions were incorporated to balance the system’s charge. Before commencing the actual simulation, the system went through energy minimization and equilibration in three stages. First, we carried out 50,000 steps of energy minimization using the steepest-descent algorithm, which stopped when the maximum force dropped below 1000 kJ/mol. Then, a 50,000-step pre-equilibration process was carried out using the NVT ensemble at 310 K, followed by another 50,000-step pre-equilibration under the NPT ensemble at 310 K and 1 atm. A 2 fs time step was used throughout these pre-simulation steps. After that, an unconstrained 100 ns simulation was run with a 2 fs time step, and the structural coordinates were saved every 10 ps. We then analyzed the simulation trajectories of the complexes formed by JAK3, PIK3CG, and SRC proteins with Peiminine.

### 4.6. Survival Analysis

To explore the association between hub genes and the overall survival of lung cancer patients, we turned to GEPIA2 [50]. We plotted Kaplan–Meier (K–M) survival curves using the LUAD (Lung adenocarcinoma) and LUSC (Lung squamous cell carcinoma) databases on this platform. We took the top three genes with the lowest binding energy from the molecular docking results and combined them with the genes related to the survival of lung cancer patients. This integrated set of genes was then used for subsequent in-depth analysis.

### 4.7. Correlation and Expression Analysis

After log2 transformation, GEPIA analyzed the correlation according to genes selected from survival analysis and docking. GEPIA generates dynamic gene expression profiles per user settings. It also compared lung cancer (LUAD and LUSC) tumor and adjacent normal sample expression profiles in the TCGA database, with *p* < 0.05 for statistical significance.

### 4.8. Validation on H1299 Cells

#### 4.8.1. Cell Culture and Peiminine Administration

The H1299 cell line was graciously supplied by Harbin Medical University. It was cultured in DMEM medium containing 1% streptomycin and penicillin, along with 10% FBS supplementation. The cells were incubated in an environment of 37 °C with 5% CO_2_. Peiminine (with a purity of at least 98%) was procured from Yuanzhi Co., Ltd. (Shanghai, China). It was dissolved in DMSO at 100 mM stored at −20 °C.

#### 4.8.2. Isolation of RNA and Reverse Transcription–Polymerase Chain Reaction

In short, following the manufacturer’s guidelines, total RNA was isolated from H1299 cells. With random primers, the extracted RNA was then reverse-transcribed into cDNA, and a qPCR assay was performed. During the detection process, the level of each sample was normalized to that of β–actin. The primer sequences for each gene are shown in Table 3.

### 4.9. Statistical Analysis

We utilized Graphpad Prism 8.0 to conduct all data analyses. To evaluate the differences between the experimental groups, we applied Student’s *t*-test and one-way ANOVA. The results were presented in the form of mean ± SEM. Statistical significance was set at * *p* < 0.05 and ** *p* < 0.01.

## 5. Conclusions

In this study, we comprehensively investigated the anti-lung cancer mechanism of peiminine. The results show that *Fritillaria ussuriensis* Maxim. from Mudanjiang has the highest peiminine content. Peiminine exerts its anti-cancer effect by modulating the PI3K–Akt signaling pathway and apoptosis-related genes. The inhibitory activity of peiminine on H1299 cell viability was validated in vitro, with underlying mechanisms involving the suppressing of the PI3K–Akt pathway. These findings indicate that peiminine has significant anti-lung cancer potential, and the targets may serve as biomarkers. Overall, this study paves the way for further research on peiminine in lung cancer treatment.

## Figures and Tables

**Table 1:** Determination of peiminine in *Fritillaria ussuriensis* Maxim. extract from 11 sources.

	Source	Content (µg)
F1	Tieli, Heilongjiang Province	457.08
F2	Tanglinnongchang, Heilongjiang Province	1023.88
F3	Mudanjiang, Heilongjiang Province	1134.64
F4	Yanshou, Heilongjiang Province	656.24
F5	Jilin Province	711.88
F6	Hebei Province	726.96
F7	Liaoning Province	730.08
F8	Changbaishan, Jilin Province	1110.72
F9	Shanghai	711.88
F10	Hubei Province	316.68
F11	Sichuan Province	933.92

**Table 2:** The docking results of 36 core genes.

Target	Uniprot ID	PDB ID	Binding Energy (kcal/mol)
PIK3CG	P48736	1E8Y	−10.1
SRC	P12931	1FMK	−10.1
JAK3	P52333	5LWM	−9.9
AKT3	Q9Y243	2X18	−9.8
MDM2	Q00987	5C5A	−9.8
IGF1R	P08069	1P4O	−9.5
MET	P08581	4R1V	−9.4
CCNA2	P20248	4EOJ	−9.3
SIRT1	Q96EB6	4KXQ	−9.3
IGF1	P05019	1TGR	−9.2
PIK3R1	P42336	7JIU	−9.2
RARA	P10276	1DSZ	−9.0
JAK2	O60674	7LL4	−9.0
RARB	P10826	4DM6	−9.0
STAT3	P40763	6NJS	−9.0
PTPN11	Q06124	3ZM1	−8.9
ERBB4	Q15303	2R4B	−8.8
KDM1A	O60341	7JK7	−8.8
MAPK14	Q16539	3LFF	−8.8
CDK4	P11802	6P8E	−8.6
AKT2	P31751	1O6L	−8.4
CDK2	P24941	1GZ8	−8.4
MAP2K1	Q02750	3EQC	−8.4
CDK1	P06493	6GU2	−8.3
EGFR	P00533	5UG9	−8.3
HDAC1	Q13547	4BKX	−8.2
HSP90AA1	P07900	5J2X	−7.9

**Table 3:** The primer sequences.

Primer Name	Sequences (5’ to 3’)
Human actin F	TCCTTCCTGGGCATGGAGT
Human actin R	AGCACTGTGTTGGCGTACAG
Human PI3K F	GGTTTGGCCTGCTTTTGGAG
Human PI3K R	CCATTGCCTCGACTTGCCTA
Human AKT F	TCTATGGCGCTGAGATTGTG
Human AKT R	CTTAATGTGCCCGTCCTTGT
Human BCL2F	GAACTGGGGGAGGATTGTGG
Human BCL2 R	CATCCCAGCCTCCGTTATCC
Human BAX F	TCAGGATGCGTCCACCAAGAAG
Human BAX R	TGTGTCCACGGCGGCAATCATC
Human PTEN F	TAGAGGAGCCGTCAAATC
Human PTEN R	ATCAGAGTCAGTGGTGTC

## Data Availability

Data are contained within the article. The data presented in this study can be requested from the authors.

## Supplementary Materials

The following supporting information can be downloaded at: https://www.mdpi.com/article/10.3390/ijms26083506/s1.
