# Supplementary material for: A Multi-Level Study on the Anti-Lung Cancer Mechanism of Peiminine, a Key Component of Fritillaria ussuriensis Maxim.: Integrating Quality Analysis, Network Pharmacology, Bioinformatics Analysis, and Experimental Validation
Source: Int J Mol Sci. 2025 Apr 9;26(8):3506. doi: 10.3390/ijms26083506 (PMC12027391; doi:10.3390/ijms26083506)
Supplement: Supplementary file 1 [file ijms-26-03506-s001.zip › ijms-3525333-supplementary.pdf]

A Multi-Level Study on the Anti-Lung Cancer Mechanism of Peiminine, a Key Component of *Fritillaria ussuriensis* Maxim.: Integrating Quality Analysis, Network Pharmacology, Bioinformatics Analysis, and Experimental Validation

Ziwen Yang [1,†], Shah Syed Faizan Ali [1,†], Xinhui Huang [1], Lin Wei [1], Yinze Zhong [1], Xuepeng Shi [1], Xiaotian Wu [1], Chunli Gan [1], Zhibin Wang [2] and Chunjuan Yang [1,*]

[1] Department of Pharmaceutical Analysis and Analytical Chemistry, College of Pharmacy, Harbin Medical University, Harbin 150000, China; ziwenyang0628@163.com (Z.Y.); fazanshah661@gmail.com (S.S.F.A.); huangxinhuiapply@163.com (X.H.); 15648571110@163.com (L.W.); zzz1170214@163.com (Y.Z.); sxp08031026@163.com (X.S.); wuxiaotian0918@126.com (X.W.); chunligan@126.com (C.G.)

[2] Key Laboratory of Basic and Application Research of Beiyao, Ministry of Education, Heilongjiang University of Chinese Medicine, Harbin 150000, China; wzbmailbox@126.com

[*] Correspondence: chunjuanyang@hrbmu.edu.cn

[†] These authors contributed equally to this work.

Supplementary Information

S1. Instruments and UHPLC-MS/MS conditions

The analysis was performed on an Agilent 1290 UHPLC system coupled with an Agilent 6430 triple quadrupole (QqQ) mass spectrometer with an electrospray ionization (ESI) source interface (Agilent Technologies, Santa Clara, CA, USA). Additionally, quantification was performed with a Waters HSS T3 column (100×2.1, 120mm, 1.8 μm). The mobile phase was Acetonitrile - 10mmol ammonium formate aqueous solution formate (65:35, v/v) at a flow rate of 0.35 mL/min. The column temperature was set at 30°C, and each injection volume was 2 μL accompanied with a needle wash process. Samples were analyzed in positive ion mode. The mass spectrum parameters were as follows: capillary voltage 4500 V; source temperature 100 ℃; desolvation temperature 350 ℃. N_2 was selected as drying gas at a flow rate of 11 L/min and high-purity N_2 was used as the nebulizing gas. The experimental data were collected by Agilent Mass Hunter workstation. The quantification was obtained in multiple reaction monitoring (MRM) mode with the precursor and product ion transitions at m/z 430.4 → 412.3 for peiminine. The fragmentor voltage (FV) was set at 130 V and collision energy (CE) at 35 eV.

S2. Method validation

The validation of our LC-MS/MS method strictly followed FDA guidelines. At least five working solutions of different concentrations were analyzed (performed in triplicate) and a calibration curve was calculated. The standard peiminine solution was further diluted to a series of concentrations to obtain the limit of quantification (LOQ), which was determined at a signal-to-noise ratio of approximately 10. The precision of the quantitative method was studied by measuring the analyte six times in one day and evaluated by the RSD value of the peak area. Repeatability was evaluated using the RSD of six independent analytical sample

solutions prepared according to the method described above. Accurately add the reference substance to the sample with known content, record the peak area, and calculate the recovery rate and RSD value. The recovery was expressed as: Recovery = (measured content—the known content)/added content of reference substance × 100%.
